# Among patients undergoing induction who reach active phase, does prolonged latent phase matter?

**DOI:** 10.1080/14767058.2025.2589629

**Published:** 2025-11-19

**Authors:** Kira A. Bromwich, Rebecca F. Hamm, Lisa D. Levine, Jennifer A. McCoy

**Affiliations:** a Department of OB/GYN, Perelman School of Medicine, University of Pennsylvania, Philadelphia, PA, USA; b Pregnancy and Perinatal Research Center, Department of OB/GYN, University of Pennsylvania Perelman School of Medicine, Philadelphia, PA, USA; c Leonard Davis Institute of Health Economics, University of Pennsylvania, Philadelphia, PA, USA

**Keywords:** Labor, latent, active, prolonged, induction, cesarean section

## Abstract

**Objective:**

We sought to assess whether a prolonged latent phase is associated with increased risk of cesarean delivery (CD) and adverse outcomes among patients undergoing induction of labor (IOL) reaching the active phase.

**Study design:**

This was a secondary analysis of a prospective cohort study from 2018–2022 of term, singleton gestations with intact membranes and unfavorable cervix undergoing IOL at two large academic hospitals. Patients who reached active labor (≥6cm dilation) were included. Prolonged latent phase was defined as <6 cm dilated after ≥12 h with ruptured membranes and oxytocin. The primary outcome was CD after reaching active phase. Secondary outcomes included CD for labor arrest and maternal/neonatal morbidity. Analyses were stratified by parity and mode of delivery, and multivariable logistic regression was performed to adjust for confounders.

**Results:**

Of 8,509 patients, 7,451 (87.6%) reached active phase. Among them, 472 (6.3%) had a prolonged latent phase, with a median duration of 15.4 h [13.3–26.8]. 74.8% of patients who reached active labor after a prolonged latent phase delivered vaginally, though they had a higher rate of CD than those without a prolonged latent phase (25.2% vs 9.9%, *p* < 0.001). This remained significant after adjusting for confounders (aRR 1.69 95%CI [1.41–2.01]). Stratifying by parity, only nulliparous patients with a prolonged latent phase remained at increased risk for CD (aRR 1.67 95%CI [1.39–2.00]). Patients with a prolonged latent phase were more likely to have clinical chorioamnionitis (20.3% vs. 9.4%, *p* < 0.001) and to experience the composite maternal and neonatal morbidities (26.9% vs. 15.1%, *p* < 0.001; 4.2% vs. 2.3%, *p* = 0.01). Among patients with a vaginal delivery, a prolonged latent phase was associated with higher maternal morbidity. However, patients who underwent CD had the highest risk of morbidity, regardless of latent phase duration.

**Conclusion:**

Among term patients undergoing induction with an unfavorable cervix, patients with a prolonged latent phase who ultimately enter active labor have a high rate of vaginal delivery. However, a prolonged latent phase is associated with increased morbidity.

## Introduction

As rates of induction of labor (IOL) in the United States continue to increase and protocols for successful induction are optimized, it is important to clearly define what it means for an induction course to be abnormal, and to set appropriate patient expectations. Specifically, standardized guidelines for diagnosing a failed IOL have become essential to clinical practice. While no universal definition exists, evidence-based guidelines generally recommend diagnosing a failed induction as the failure to achieve active labor (in current practice, defined as ≥ 6cm dilation) after 12 to 24 hours of oxytocin use in the setting of rupture of membranes [1–3]. An evidence-based definition for failed IOL is critical to help balance maximizing the opportunity for patients to achieve a vaginal delivery, without fruitlessly incurring the maternal and neonatal risks associated with a prolonged labor course.

However, little is known about the potential associations of a prolonged, but not failed, latent phase of labor. Studies have shown increased rates of chorioamnionitis, endometritis, and postpartum hemorrhage after prolonged latent phase [2–6]. Importantly, these studies have primarily included those who were diagnosed with a failed IOL and underwent cesarean delivery (CD), and have also included patients with a prolonged latent phase in spontaneous labor [7–9]. There is relatively little evidence specifically examining risks and outcomes for those who have a prolonged latent phase after IOL but do ultimately still progress [10]. More data is needed to understand the clinical implications of a prolonged latent phase among those patients who do ultimately reach the active phase of labor. This knowledge is critical not just because prolonged latent phase has been linked to adverse maternal and neonatal outcomes, but also to inform and individualize patient counseling and management for the duration of the labor course [9,11]. Therefore, we sought to assess, among patients undergoing IOL who reach the active phase of labor, whether a prolonged latent phase is associated with an increased risk of CD and increased risk of maternal and neonatal adverse outcomes.

## Materials and methods

This was a secondary analysis of a prospective study examining implementation of a standardized labor induction protocol at two large academic hospitals from 2018 to 2022 [12]. The study utilized an eight-component protocol centered on active management of induction of labor, which included frequent cervical exams and interventions such as oxytocin and amniotomy (see Supplemental Figure S1 for full standardized protocol for latent and active labor) [12,13]. The project was approved by the University of Pennsylvania institutional review board as quality improvement with a waiver of informed consent in April, 2020. The parent study included 8,509 patients who met the following inclusion criteria: term (≥37 weeks) IOL for any indication, singleton gestation in cephalic presentation, intact membranes, and an unfavorable cervix (defined as Bishop score of ≤6 and dilation ≤2.) Those with a prior cesarean delivery were excluded. For the purposes of this secondary analysis, only participants who reached the active phase of labor were included, as outcomes for those with a CD in latent phase are well documented [3]. Active phase was defined as achieving ≥6 cm cervical dilation [14]. Prolonged latent phase was defined as <6 cm dilation after ≥12 h with ruptured membranes and receiving oxytocin infusion [15]. These criteria were selected so as to approach, but not exceed, the lower limit of accepted definitions for failed induction of labor [16]. Checks were conducted at standard intervals in all patients. The primary outcome was CD for any indication after reaching the active phase. Secondary outcomes included CD for labor arrest (including CD performed for arrest of dilation in active phase, arrest of descent, or failed operative vaginal delivery), length of active phase, length of second stage, and active phase dystocia (defined as having the same cervical dilation over ≥2 h in active phase). Additional secondary outcomes included composite as well as individual maternal and neonatal morbidities. Composite maternal morbidity was defined as ≥1 of the following: endometritis, postpartum hemorrhage (defined as estimated blood loss >1000 ml), blood transfusion, wound infection, venous thromboembolism, hysterectomy, intensive care unit admission, readmission within 30 days, and death. Additional maternal morbidity outcomes evaluated were clinical chorioamnionitis and higher order laceration (3a or higher). Clinical chorioamnionitis and endometritis diagnoses were confirmed with record review. Composite neonatal morbidity was defined as ≥1 of the following: severe respiratory distress (defined as intubation and mechanical ventilation for a minimum of 12 h) and culture-proven sepsis requiring antibiotics. Additional neonatal outcomes evaluated were birth weight at delivery, 5-min Apgar score, neonatal intensive care unit (NICU) admission, NICU admission >48 h, and neonatal length of stay.

Bivariate analyses were performed using chi-square and Fisher’s exact tests to compare categorical data, and student’s t-tests and Mann–Whitney U to compare parametric and nonparametric data. Relative risk (RR) of cesarean delivery was estimated with a modified Poisson’s regression model with robust error variance. Factors with *p* < 0.10 in bivariate analysis were evaluated as possible confounders in the regression model; these included maternal BMI, parity, insurance coverage, pre-gestational or gestational diabetes, modified Bishops’ score, and IOL indication. Backwards stepwise elimination was used to obtain a parsimonious model. Exploratory analyses were performed stratifying by parity given known differences in CD risk by parity. Based on a fixed sample size of 8,509 patients in the parent study, we assumed 80% would reach active phase, of whom 5% would meet criteria for prolonged labor, and we would then have 90% power to detect a 1.4-fold difference in CD risk. All data were analyzed using Stata version 15.0 (College Station, TX). A *p* value <0.05 was considered statistically significant.

## Results

Of 8,509 patients in the cohort, 7,451 (87.6%) reached active phase (≥6 cm cervical dilation) and were therefore included in this secondary analysis. Of those, 472 (6.3%) patients met criteria for a prolonged latent phase. As shown in Table 1, 43.3% of patients self-identified as Black, 39.2% were publicly insured, and 61.8% were nulliparous. There were no significant differences in race, age, gestational age at delivery, or presence of maternal hypertension between patients with and without a prolonged latent phase. Patients with a prolonged latent phase had a higher body mass index (BMI), and were more likely to be nulliparous, privately insured, and to have gestational or pre-gestational diabetes. They also had a lower Bishop score at the start of IOL and differences in indication for induction and induction method utilized (Table 1).

As shown in Table 2, the median number of hours in latent labor with ruptured membranes and receiving oxytocin was 15.4 h [13.3–26.9] in the prolonged group, vs. 3.8 h [1.4–6.7] in the normal latent phase group. In regards to the primary outcome, the majority of patients with a prolonged latent phase did ultimately achieve a vaginal delivery (*n* = 353, 74.8%). However, those with prolonged latent phase had a higher rate of CD than those without a prolonged latent phase (25.2% vs 9.9%, *p* < 0.001). This difference persisted when adjusting for confounders including BMI, insurance status, diabetes, and Bishop score at the start of induction (aRR 1.69 95%CI [1.41–2.01]). Among 4,607 nulliparous patients, 410 (8.9%) had a prolonged latent phase while among 2,844 multiparous patients, 62 (2.1%) had a prolonged latent phase. When stratifying by parity, the adjusted risk of CD remained significant only among nulliparous patients who reached active phase (aRR 1.67 95%CI [1.39–2.00]), but not among multiparous patients who reached active phase (aRR 2.13 95%CI [0.88–5.12]). Furthermore, among nulliparous patients, there was a higher rate of CD for a labor arrest indication among patients with prolonged latent phase compared to those without (19.5% vs. 6.3%, *p* < 0.001). The overall median duration of both the active phase and the second stage of labor was significantly longer in those with versus without a prolonged latent phase: active phase: 2.0 h [0.0–4.5] vs. 1.0 h [0.0–3.0], *p* < 0.001; second stage: 1.7 h [0.7–2.4] vs. 0.7 h [0.3–1.7], *p* < 0.001. Additionally, those with a prolonged latent phase had a higher rate of active phase dystocia compared to those without a prolonged latent phase (13.1% vs. 8.5%, *p* = 0.001).

**Figure 1.**
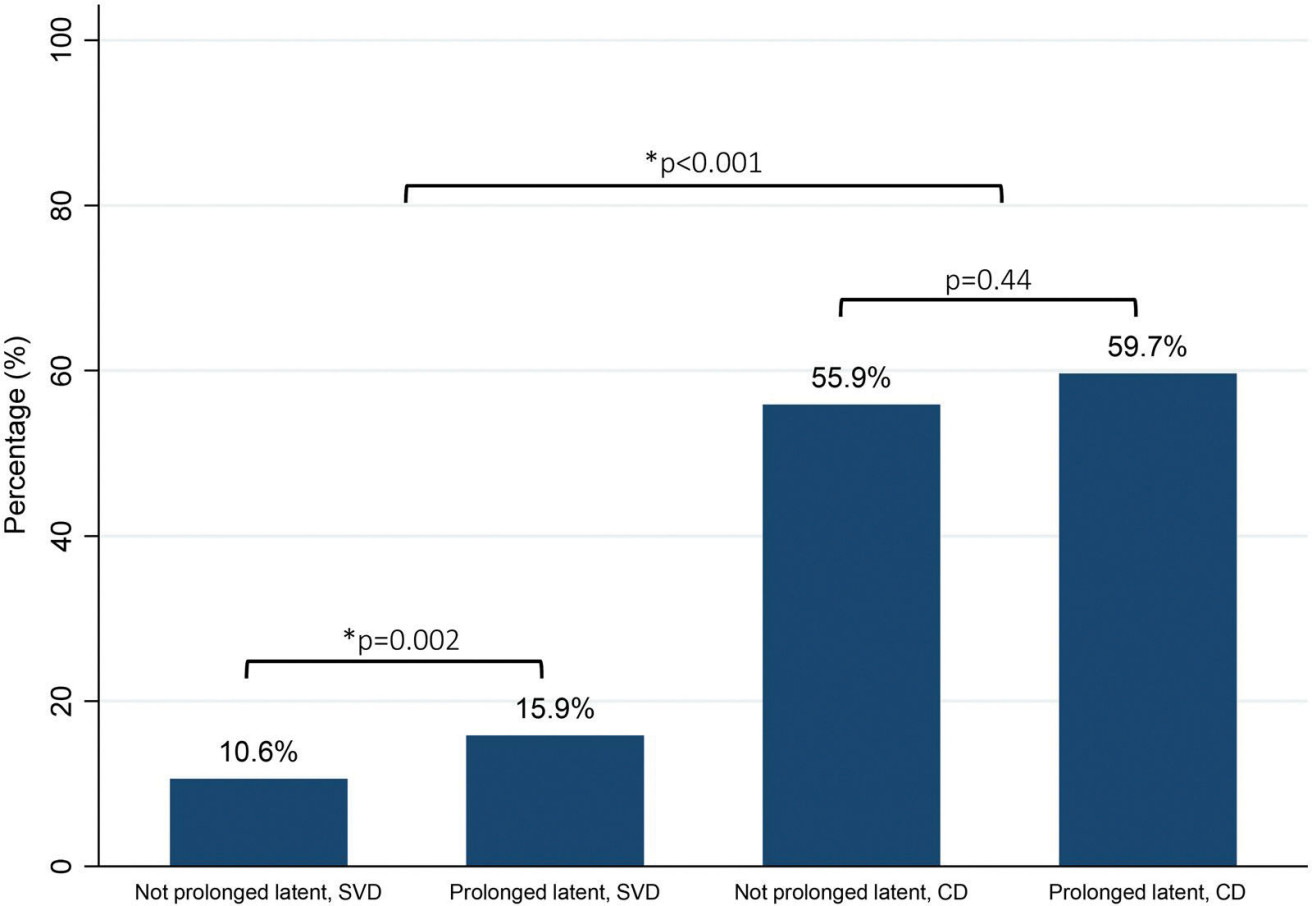
Composite maternal morbidity by prolonged vs. not prolonged latent phase and mode of delivery. Abbreviations. SVD: vaginal delivery; CD: cesarean delivery.

Next, we assessed maternal and neonatal morbidity. There were higher rates of maternal and neonatal morbidities in the prolonged latent phase group, as compared to those without prolonged labor (Table 3). Those with a prolonged latent phase were more likely to be diagnosed with clinical chorioamnionitis (20.3% vs. 9.4%, *p* < 0.001), and to experience the composite maternal morbidity (26.9% vs. 15.1%, *p* < 0.001). Babies born to patients with a prolonged latent phase were more likely to experience the composite neonatal morbidity (4.2% vs. 2.3%, *p* = 0.01). We then stratified this analysis by mode of delivery. Table 4 displays morbidity data in groups compared between those with and without prolonged latent phase, as well as mode of delivery (vaginal vs. CD). Among those who had a vaginal delivery, patients with a prolonged latent phase had significantly increased rates of both maternal and neonatal morbidity outcomes, as noted in Table 4. However, among those who had a CD, when comparing those with and without prolonged latent labor, the only difference in morbidity outcomes was in the risk of endometritis. Figure 1 depicts composite maternal morbidity by prolonged latent phase and mode of delivery, demonstrating that the highest rates of morbidity were among those who underwent CD, regardless of whether they had prolonged latent phase.

## Discussion

Our findings demonstrate that the vast majority of patients undergoing IOL who have a prolonged latent phase will go on to have a vaginal delivery. However, a prolonged latent phase is associated with an increased risk of maternal and neonatal morbidity, even among those who ultimately experience a vaginal delivery. While these findings provide reassurance for both providers and patients that a vaginal delivery remains the most likely outcome, even after a prolonged latent phase, the increased risks of maternal and neonatal morbidity should be discussed when a prolonged latent phase occurs.

Comparability with prior studies is limited given the paucity of data addressing our specific population of interest, widely varying definitions of prolonged latent labor, and the evolution of the definition of active labor. Even so, our findings are consistent with those of Rouse et al. Kawakita et al. Simon et al. and Grobman et al. that while a prolonged latent phase is associated with increased risk of CD, a substantial portion of these patients go on to have a vaginal delivery [1–3,5,6]. Of these, only Kawakita and Rouse’s studies included both nulliparous and multiparous patients [1,5]. Our study echoes their conclusion that even following a prolonged latent phase, vaginal delivery rates remain higher among multiparas than nulliparas.

Previous studies have also demonstrated that a prolonged latent phase is associated with an increased risk of maternal adverse outcomes; specifically, maternal EBL, rates of chorioamnionitis, and endometritis, have all been shown to increase with a prolonged latent phase [2,3, 5,6, 9–11,17,18]. In all five studies focused on induction either exclusively or in a subgroup analysis, increased maternal morbidity was driven by either infection [5], postpartum hemorrhage [10], or both [2,3,6]. There is a lack of consensus regarding the impact of a prolonged latent phase on the rate of adverse neonatal outcomes, which our study did find; this may be due to the aforementioned high degree of variation in the definitions used in prior studies [5,9].

One recent study by Burd et al. shares our specific population of interest – nulliparous and multiparous patients with a prolonged latent phase who reach the active phase [10]. They demonstrated high rates of vaginal delivery, increased rates of maternal morbidity only among those who underwent CD, with no difference among those who achieved SVD. This contrasts with our results, which showed more discrepant morbidity as a function of prolonged latent phase in patients who had a vaginal delivery. It is also notable that our maternal morbidity rates were higher (26.9% in induced patients with a prolonged latent phase versus 17.7%.) This is possibly explained by several factors. While Burd et al. did stratify by spontaneous and induced labor, their sub-analysis of mode of delivery did not distinguish between these two groups. Furthermore, our definitions of prolonged latent labor varied significantly: >90%ile between admission and active phase in their study – or 18.75 h in induced patients – versus ≥12 h with ruptured membranes and oxytocin in ours. Importantly, our studies are consistent in that regardless of length of latent phase, the highest morbidity is among patients who undergo CD.

Indeed, other studies confirm that among patients with prolonged latent phase, the highest rates of maternal morbidity occur in patients who undergo CD [1,3,9]. While Chelmow et al. focused exclusively on patients in spontaneous labor, they demonstrated an over 25% rate of febrile maternal morbidity in patients with a prolonged latent phase undergoing CD, which is comparable to our study [9]. Additionally, data supports high rates of maternal morbidity associated with CD for arrest of active phase, similar to the rates observed in our study [19,20]. Our findings add to the literature the distinction that while a prolonged latent phase does confer some increased risk of maternal morbidity, mode of delivery remains the more significant mediator of morbidity, and a vaginal delivery remains the safest outcome.

### Strengths and limitations

This study has several notable strengths including size of the population, inclusion of nulliparous and multiparous patients alike, and spanning various maternal and fetal indications for induction. The racially diverse makeup of our study population is an added strength, particularly as Black patients have historically been underrepresented in obstetric research and are known to be at higher risk for adverse pregnancy outcomes. Our limitations must be acknowledged as well. Because the study was conducted across two sites within a single institution, findings may not be generalizable across varied patient populations and practice settings. Finally, our study is limited by the fact that there is no universally accepted definition for a prolonged latent phase of labor, though concurrence with our findings among studies using different time cutoffs suggests that our findings can be generalized to include longer latent phases.

## Conclusions

Our findings have demonstrated that in this single center study, while patients with prolonged latent phase prior to reaching the active phase of labor are at increased risk of CD, the majority still went on to deliver vaginally. After adjusting for confounders, a prolonged latent phase in multiparous patients who ultimately reach active labor is not associated with increased risk of CD. Morbidity is increased after a prolonged latent phase, but CD remains the biggest driver of both maternal and neonatal morbidities. These findings can help inform patient and provider expectations regarding the expected labor course after a prolonged latent phase and provide reassurance that the risks of an active phase CD are overall not substantially increased after a prolonged latent phase. Future research might investigate how various labor management strategies can mediate latent labor length, further optimize vaginal delivery rates, and reduce risk, particularly in nulliparous patients.

## Supplementary Material

Supplementary Figure

Supplemental data for this article can be accessed online at https://doi.org/10.1080/14767058.2025.2589629.

## Figures and Tables

**Table 1.** Demographic and clinical baseline characteristics in patients with a normal versus prolonged latent phase of labor.

	Normal latent phase (*n* = 6979)	Prolonged latent phase (*n* = 472)	*p* Value

Race			0.13
Black	3042 (43.6%)	188 (39.8%)	
White	2883 (41.3%)	200 (42.4%)	
Other^a^	1054 (15.1%)	84 (17.8%)	
Maternal (years)^b^	31.2 [26.7–34.6]	31.1 [25.2–34.6]	0.40
BMI (mg/kg2)^b^	31.2 [27.6–36.2]	33.2 [29.0–38.3]	<0.001
Nulliparous	4197 (60.1%)	410 (86.9%)	<0.001
Insurance			<0.001
Private	4189 (60.1%)	321 (68.2%)	
Public	2777 (39.9%)	150 (31.8%)	
Gestational diabetes	540 (7.8%)	54 (11.4%)	0.010
Pre-gestational diabetes	628 (9.0%)	64 (13.6%)	<0.001
Chronic hypertension	567 (8.1%)	45 (9.6%)	0.49
Nulliparity	4197 (60.1%)	410 (86.9%)	<0.001
Bishop score at start of induction^b^	2.0 [1.0–3.0]	2.0 [0.0–2.0]	<0.001
Dilation at start of induction			<0.001
0 cm	2138 (30.6%)	242 (51.3%)	
1–2 cm	4566 (65.4%)	224 (47.5%)	
3–4 cm	275 (3.9%)	6 (1.3%)	
Gestational age at delivery (weeks)^b^	39.5 [38.7–40.3]	39.6 [38.5–40.4]	0.51
Epidural use	6492 (93.0%)	462 (97.9%)	<0.001
Indication for induction			<0.001
Elective/postdates	2334 (33.4%)	127 (26.9%)	
Maternal	2402 (34.4%)	207 (43.9%)	
Fetal	1295 (18.6%)	77 (16.3%)	
Other	948 (13.6%)	61 (12.9%)	
IOL agent			<0.001
Misoprostol alone	1170 (16.8%)	54 (11.4%)	
Misoprostol + Foley balloon (FB)	3449 (49.4%)	194 (41.1%)	
FB alone	250 (3.6%)	15 (3.2%)	
FB + Oxytocin	419 (6.0%)	41 (8.7%)	
Other	10 (0.1%)	1 (0.2%)	

a Other category includes American Indian or Alaska Native, Asian, Native Hawaiian or Other Pacific Islander, and other as designated by patient.

b All data presented as n(%) unless otherwise noted. b. median [IQR]. BMI: body mass index.

**Table 2.** Maternal labor outcomes.

	Normal latent phase (*n* = 6979)	Prolonged latent phase (*n* = 472)	*p* Value

Length of time in latent phase with ruptured membranes and receiving oxytocin (hours)	3.8 [1.4, 6.7]	15.4 [13.3, 26.8]	<0.001
Length of total latent phase, including cervical ripening^a^ (hours)	13.3 (9.4, 19,0)	27.6 (22.6, 33.9)	<0.001
Length of active phase^a^ (hours)	1.0 (0.0, 3.0)	2.0 (0.0, 4.5)	<0.001
Length of active phase (hours)			
0	2,249 (32.2)	121 (25.6)	<0.001
0–2	2,242 (32.1)	118 (25.0)	
2–4	1,207 (17.3)	92 (19.5)	
4–6	539 (7.7)	67 (14.2)	
>6	742 (10.63)	74 (15.7)	
Length of second stage^a^ (hours)	0.7 (0.3, 1.7)	1.7 (0.7, 2.4)	<0.001
Length of total labor^a^ (hours)	16.3 (11.3, 23.0)	32.1. (26.5, 39.2)	<0.001
Vaginal delivery	6290 (90.1%)	353 (74.8%)	<0.001
Cesarean delivery	689 (9.9%)	119 (25.2%)	<0.001
Cesarean by parity:			
Nulliparous (*n* = 714)	600 (14.3%)	114 (27.8%)	<0.001
Multiparous (*n* = 94)	89 (3.2%)	5 (8.1%)	0.03
indication for cesarean (*n* = 808):			<0.001
Labor arrest indication	437 (63.4%)	92 (77.3%)	
NRFHT	222 (32.2%)	22 (18.5%)	
Other indication	30 (4.4%)	5 (4.2%)	

a All data presented as n(%) unless otherwise noted. a. median [IQR].

**Table 3.** Maternal and neonatal morbidity.

	Normal latent phase (*n* = 6979)	Prolonged latent phase (*n* = 472)	*p* Value

Maternal outcomes:			
Postpartum hemorrhage (EBL > 1000 mL)	607 (8.9%)	88 (18.8%)	<0.001
Maternal blood transfusion	176 (2.5%)	31 (6.6%)	<0.001
Clinical chorioamnionitis	659 (9.4%)	96 (20.3%)	<0.001
Postpartum endometritis	67 (1.0%)	14 (3.0%)	<0.001
Wound separation or infection	119 (1.7%)	13 (2.8%)	0.09
ICU admission	18 (0.3%)	1 (0.2%)	1.00
Readmission within six weeks	148 (2.1%)	16 (3.4%)	0.07
Composite maternal morbidity	1052 (15.1)	127 (26.9)	<0.001
Neonatal outcomes:			
Birth weight at delivery^a^	3270 (2950, 3570)	3307 (3050, 3620)	0.02
Apgar 5 min^a^	9.0 (9.0, 9.0)	9.0 (9.0, 9.0)	<0.001
NICU admission	761 (10.9%)	86 (18.3%)	<0.001
NICU admission > 48 h	328 (4.7%)	30 (6.4%)	0.08
Severe respiratory distress	156 (2.2%)	20 (4.2%)	0.03
Neonatal sepsis			
Presumed	409 (5.9%)	65 (13.8%)	<0.001
Culture proven	7 (0.1%)	0 (0.0%)	0.51
Composite neonatal morbidity	162 (2.3)	20 (4.2)	0.01

a All data presented as n(%) unless otherwise noted. a. median [IQR]. Composite maternal morbidity defined as: ≥1 of the following: endometritis, postpartum hemorrhage, blood transfusion, wound infection, venous thromboembolism, hysterectomy, intensive care unit admission, readmission within 30 days, and death. Composite neonatal morbidity defined as: ≥1 of the following: severe respiratory distress and culture-proven sepsis requiring antibiotics.

Abbreviations. EBL, estimated blood loss; ICU, intensive care unit; NICU, neonatal intensive care unit.

**Table 4.** Maternal and neonatal morbidity and mode of delivery.

	Not-prolonged SVD (*n* = 6290)	Prolonged SVD (*n* = 353)	*p* Value	Not-prolonged CD (*n* = 689)	Prolonged CD (*n* = 119)	*p* Value

Maternal outcomes:						
Postpartum hemorrhage (EBL > 1000 mL)	258 (4.2%)	28 (8.0%)	<0.001	349 (50.7%)	60 (50.4%)	0.95
Maternal blood transfusion	120 (1.9%)	19 (5.4%)	<0.001	56 (8.1%)	12 (10.1%)	0.48
Clinical chorioamnionitis	490 (7.8%)	60 (17.0%)	<0.001	169 (24.5%)	36 (30.3%)	0.19
Postpartum endometritis	48 (0.8%)	3 (0.8%)	0.86	19 (2.8%)	11 (9.2%)	<0.001
Wound separation or infection	90 (1.4%)	8 (2.3%)	0.21	29 (4.2%)	5 (4.2%)	1.00
ICU admission	10 (0.2%)	0 (0.0%)	1.00	8 (1.2%)	1 (0.8%)	1.00
Readmission within six weeks	130 (2.1%)	12 (3.4%)	0.09	18 (2.6%)	4 (3.4%)	0.64
Composite maternal morbidity	667 (10.6%)	56 (15.9%)	0.002	385 (55.9%)	71 (59.7%)	0.44
Neonatal outcomes:						
Apgar 5 min^a^	9.0 (9.0, 9.0)	9.0 (9.0, 9.0)	<0.001	9.0 (9.0, 9.0)	9.0 (9.0, 9.0)	0.42
NICU admission	621 (9.9%)	62 (17.6%)	<0.001	140 (20.3%)	24 (20.2%)	0.97
NICU admission >48 h	277 (46.2%)	23 (37.1%)	0.17	52 (38.1%)	7 (29.2%)	0.41
Severe respiratory distress	122 (1.9%)	15 (4.2%)	0.02	34 (4.9%)	5 (4.2%)	1.00
Neonatal sepsis						0.13
Presumed	333 (5.3%)	44 (12.5%)	<0.001	76 (11.1%)	21 (17.6%)	
Culture proven	4 (0.1%)	0 (0.0%)		3 (0.4%)	0 (0.0%)	
Composite neonatal morbidity	125 (2.0%)	15 (4.2%)	0.004	36 (5.2%)	5 (4.2%)	0.64

a All data presented as n(%) unless otherwise noted. a. median [IQR]. Composite maternal morbidity defined as: ≥1 of the following: endometritis, postpartum hemorrhage, blood transfusion, wound infection, venous thromboembolism, hysterectomy, intensive care unit admission, readmission within 30 days, and death. Composite neonatal morbidity defined as: ≥1 of the following: severe respiratory distress and culture-proven sepsis requiring antibiotics.

Abbreviations. SVD, spontaneous vaginal delivery; CD, cesarean delivery; EBL, estimated blood loss; ICU, intensive care unit; NICU, neonatal intensive care unit.

## Data Availability

The authors confirm that the data supporting the findings of this study are available within the article [and/or] its supplementary materials.
