# Supplementary material for: Among patients undergoing induction who reach active phase, does prolonged latent phase matter?
Source: J Matern Fetal Neonatal Med. Author manuscript; Available in PMC 2026 May 8. (PMC13154326; doi:10.1080/14767058.2025.2589629)
Supplement: Supplementary Figure [file NIHMS2141170-supplement-Supplementary_Figure.docx]

Supplemental Figure 1:**** Standardized protocol for the management of latent labor. 1b: Standardized protocol for the management of active labor.
